# Transcriptomic and metabolomic analyses provide new insights into the appropriate harvest period in regenerated bulbs of *Fritillaria hupehensis*

**DOI:** 10.3389/fpls.2023.1132936

**Published:** 2023-02-15

**Authors:** Yuanyuan Duan, Jiaqi Wu, Fanfan Wang, Kaiqi Zhang, Xiaoliang Guo, Tao Tang, Sen Mu, Jingmao You, Jie Guo

**Affiliations:** ^1^ Key Laboratory of Biology and Cultivation of Chinese Herbal Medicines, Ministry of Agriculture and Rural Affairs, Institute of Chinese Herbal Medicines, Hubei Academy of Agricultural Sciences, Enshi, China; ^2^ Hubei Engineering Research Center of Under-forest Economy, Hubei Academy of Agricultural Sciences, Wuhan, China; ^3^ Hubei Engineering Research Center of Good Agricultural Practices (GAP) Production for Chinese Herbal Medicines, Institute of Chinese Herbal Medicines, Hubei Academy of Agricultural Sciences, Enshi, China

**Keywords:** *Fritillaria hupehensis*, bulb development, steroidal alkaloids, metabolic, WGCNA

## Abstract

**Introduction:**

The bulb of *Fritillaria hupehensis*, a traditional cough and expectorant medicine, is usually harvested from June to September according to traditional cultivation experience, without practical scientific guidance. Although steroidal alkaloid metabolites have been identified in *F. hupehensis*, the dynamic changes in their levels during bulb development and their molecular regulatory mechanisms are poorly understood.

**Methods:**

In this study, integrative analyses of the bulbus phenotype, bioactive chemical investigations, and metabolome and transcriptome profiles were performed to systematically explore the variations in steroidal alkaloid metabolite levels and identify the genes modulating their accumulation and the corresponding regulatory mechanisms.

**Results:**

The results showed that weight, size, and total alkaloid content of the regenerated bulbs reached a maximum at IM03 (post-withering stage, early July), whereas peiminine content reached a maximum at IM02 (withering stage, early June). There were no significant differences between IM02 and IM03, indicating that regenerated bulbs could be harvested appropriately in early June or July. Peiminine, peimine, tortifoline, hupehenine, korseveramine, delafrine, hericenone N-oxide, korseveridine, puqiedinone, pingbeinone, puqienine B, puqienine E, pingbeimine A, jervine, and ussuriedine levels were upregulated in IM02 and IM03, compared with IM01 (vigorous growth stage, early April). The Kyoto Encyclopedia of Genes and Genomes enrichment analysis indicated that the accumulation of steroidal alkaloid metabolites mainly occurred prior to IM02. *HMGR1*, *DXR*, *CAS1*, *CYP 90A1*, and *DET2* may play a positive role in peiminine, peimine, hupehenine, korseveramine, korseveridine, hericenone N-oxide, puqiedinone, delafrine, tortifoline, pingbeinone, puqienine B, puqienine E, pingbeimine A, jervine, and ussuriedine biosynthesis, whereas the downregulation of *FPS1*, *SQE* and *17-DHCR* may lead to a reduction in peimisine levels. Weighted gene correlation network analysis showed that *CYP 74A2-1, CYP 74A2-2, CYP 71A26-1*, *CYP 71A26-2*, and *CYP74A* were negatively correlated with peiminine and pingbeimine A, whereas *CYP R* and *CYP707A1* were positively correlated. *. CYP 74A2-1* and *CYP 74A2-2* may play a negative role in peimine and korseveridine biosynthesis, whereas *CYP R* plays a positive role. In addition, the highly expressed C2H2, HSF, AP2/ERF, HB, GRAS, C3H, NAC, MYB-related transcription factors (TFs), GARP-G2-like TFs, and WRKY may play positive roles in the accumulation of peiminine, peimine, korseveridine, and pingbeimine A.

**Discussion:**

These results provide new insights into scientific harvesting of *F. hupehensis*.

## 1 Introduction

*Fritillaria hupehensis* Hsiao et K.C. Hsia, a member of the Liliaceae family, is a well-known bulbus medicinal plant (Zhang et al., 2007). Dried bulbs have been clinically used as antitussives and expectorants for thousands of years (Wang et al., 2007; Zhang et al., 2008). As key Chinese medicinal materials, *F. hupehensis* bulbs have been widely cultivated in central China and increasingly exported in recent decades (Wang et al., 2021a). Despite its wide range of applications and long history of use, there have been few systematic studies on the growth and development of *F. hupehensis*. It is generally acknowledged that each herbal medicine has an appropriate harvest period (Shan et al., 2019). Based on thousands of years of traditional planting experience, the bulbs of *F. hupehensis* are often harvested from June (when the plant has fallen) to September. However, is this time an appropriate harvest period and why is this period selected? Unfortunately, these studies have not yet been conducted, and cogent scientific evidence remains limited.

The development of medicinal herbs is a complex process involving numerous physiological and structural alterations, and secondary metabolism (Huang et al., 2019; Li et al., 2020b). Secondary metabolites are crucial sources of natural medicines and form the material basis for their clinical curative effects (Li et al., 2020b; Tang et al., 2021). Moreover, steroidal alkaloids, a major class of important secondary metabolites, have been reported to be the dominant chemical constituents in *F. hupehensis* (Guo et al., 2021; Duan et al., 2022). To date, increasing attempts have been made to identify the chemical composition of *F. hupehensis*, with a particular focus on steroidal alkaloids such as peiminine, peimine, hupehenine, and peimisine (Kumar et al., 2020; Liu et al., 2021). Many clinical biochemical activity studies have been conducted in recent years (Wu et al., 2018; Zhang et al., 2021; Xiang et al., 2022). However, variations in the bioactive phytochemicals during bulb development in *F. hupehensis* remain poorly understood.

In recent years, integrative analyses of metabolic and transcriptome data have been performed to investigate secondary metabolic compositions, identify gene functions, and elucidate metabolic pathways during plant development (Li et al., 2020a; Bai et al., 2021; Zheng et al., 2021). Notably, integration efforts have been successfully conducted for the fruit development of model plants (Li et al., 2020a), fruits (Wang et al., 2016; Li et al., 2019), and nuts (Wang, H. et al., 2021; Zhao et al., 2022). Furthermore, the effective application of multiple omics analyses has been witnessed in investigating the mechanism of medicinal plant secondary metabolite biosynthesis and identifying gene functions (Xu et al., 2020; Si et al., 2022; Wu et al., 2022). Notably, the genes involved in regulating the steroidal alkaloids have not been characterized during bulb development in *F. hupehensis*, and their molecular mechanisms have not yet been elucidated.

In this study, the bulbus phenotype and bioactive chemical investigations were performed during bulb development to identify the appropriate time for harvesting *F. hupehensis*. Ultra-performance liquid chromatography-tandem mass spectrometry (UPLC-MS/MS) and RNA sequencing (RNA-Seq) analyses were used to investigate the differences in alkaloids and genes in regenerated bulbs at different stages. Co-expression analysis integrated with metabolomics and transcriptomics was conducted to systematically explore the molecular mechanisms of steroidal alkaloid biosynthesis during regenerated bulb development in *F. hupehensis*. The results provide an overview of the appropriate harvest period of *F. hupehensis* and improve our understanding of steroidal alkaloid biosynthesis mechanisms in regenerated bulb development.

## 2 Methods and materials

### 2.1 Plant materials

Phenotypic and biochemical variations in the regenerated fresh bulbs of *F. hupehensis* were investigated at five developmental stages: IM0a (seedling stage, early March), IM01 (vigorous growth stage, early April), IM0b (early withering stage, early May), IM02 (withering stage, early June), and IM03 (post-withering stage, early July). Healthy regenerated fresh bulbs were collected from *F. hupehensis*-*M. officinalis* intercropping systems. The test sample site and cropping pattern were described in detail in our previous study (Duan et al., 2022). Row spacings of *M. officinalis* trees were 1.50 m × 2.00 m. Bulbs of *F. hupehensis* were sown under *M. officinalis* trees, and their row spacings were 10 cm × 15 cm. The experimental plot size was 6.67 m^2^ with three replicates. Fresh bulbs were used immediately for phenotypic and biochemical measurements. Thereafter, bulbs collected from stages IM01, IM02, and IM03 were frozen in liquid nitrogen and stored at -80°C for further analysis.

### 2.2 Phenotypic and biochemical measurements

Bulb weight was measured using an electronic balance and bulb size (length, width, and thickness) was measured using a Vernier caliper with nine replicates. The total alkaloid content was measured using an ultraviolet spectrophotometer (UV-1800, Shimadzu, Japan) according to the method described by Zhou et al. (2022). The peiminine content was analyzed using a high-performance liquid chromatography system (Agilent 1260, Agilent, USA) according to *the Pharmacopoeia of the People’s Republic of China* (Version 2020). The total alkaloid and peiminine contents were measured using three biological replicates.

### 2.3 Alkaloid metabolomic analysis

Alkaloid extract preparation, identification, and metabolite quantification were conducted as previously described (Duan et al., 2022). Three biological replicates (three bulbs per replicate) were analyzed. Alkaloids with variable importance in projection (VIP) ≥1 and |log_2_ (fold change) |≥1 were defined as differentially expressed alkaloids (DEAs).

### 2.4 Transcriptomic analysis

Total RNA extraction, RNA integrity, and concentration determination were performed as described in our previous study (Duan et al., 2022). Subsequently, high-quality RNA was used to construct a cDNA library, which was then sequenced using an Illumina HiSeq 2500. After removing contaminating reads, invalid reads with N ratios greater than 10%, and low-quality reads, 60.18-Gb of clean reads were obtained. Clean reads were assembled *de novo* using Trinity (v2.11.0) to obtain high-quality transcript sequences (Grabherr et al., 2011). Alkaloid metabolite detection and sequencing profiles were obtained from Wuhan MetWare Biotechnology Co., Ltd. (Wuhan, China).

Gene expression levels were estimated as fragments per kilobase million (FPKM). Differentially expressed genes (DEGs) were identified using a threshold of |Log_2_ (fold change) |≥1, false discovery rate, and adjusted *p*<0.05. The Kyoto Encyclopedia of Genes and Genomes (KEGG) enrichment analysis was performed using the KEGG database.

### 2.5 Weighted gene co-expression network analysis

Weighted gene co-expression network analysis (WGCNA) was performed using R (version 1.66), based on gene expression levels (FPKM>5). Nine differentially expressed steroidal alkaloids were used as trait files to generate co-expression networks and modules. Soft-threshold Pearson’s correlations between traits and modules were calculated. KEGG enrichment analysis was performed using the Metware cloud tools (https://cloud.metware.cn/#/tools). Cystoscope (version 3.8.1) was used to visualize the co-expression network.

### 2.6 Quantitative real-time PCR analysis

Ten target genes with higher expression were randomly selected for quantitative real-time PCR (qRT-PCR) analysis, and *18S rRNA* was selected as the internal reference gene. qRT-PCR was performed as described previously (Duan et al, 2022). The relative expression levels of the test genes in the three biological replicates were calculated using 2^-ΔΔCt^. Primer sequences are listed in **Table S1**.

### 2.7 Statistical analysis

**Figure 1:**
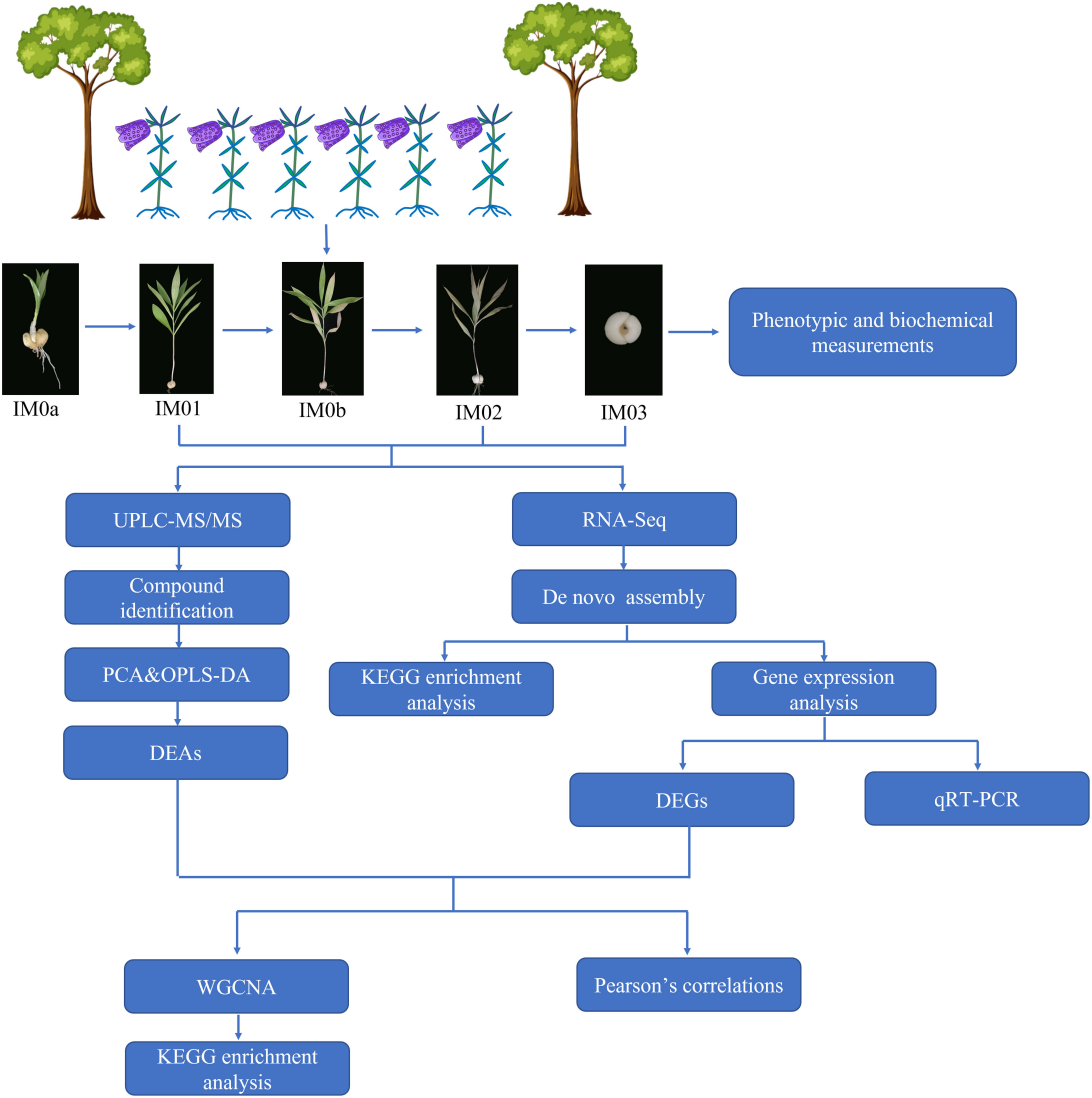
Schematic diagram of the analysis pipeline in this study.

Statistical data were analyzed using Microsoft Excel (2019). Significant differences among the samples (*p*<0.05) were examined using SPSS 19.0. Principal component analysis (PCA) and orthogonal partial least-squares discriminant analysis (OPLS-DA) were performed using R-models. Origin Pro 2021 software was used to plot graphs and analyze Pearson’s correlations among DEGs and steroidal alkaloids. Heatmaps of DEGs and DEAs were generated using the TBTools software (version 1.068). The analysis pipeline is shown in **Figure 1**.

## 3 Results

### 3.1 Characterization of dynamic variations in regenerated bulb development of *F. hupehensis*

**Figure 2:**
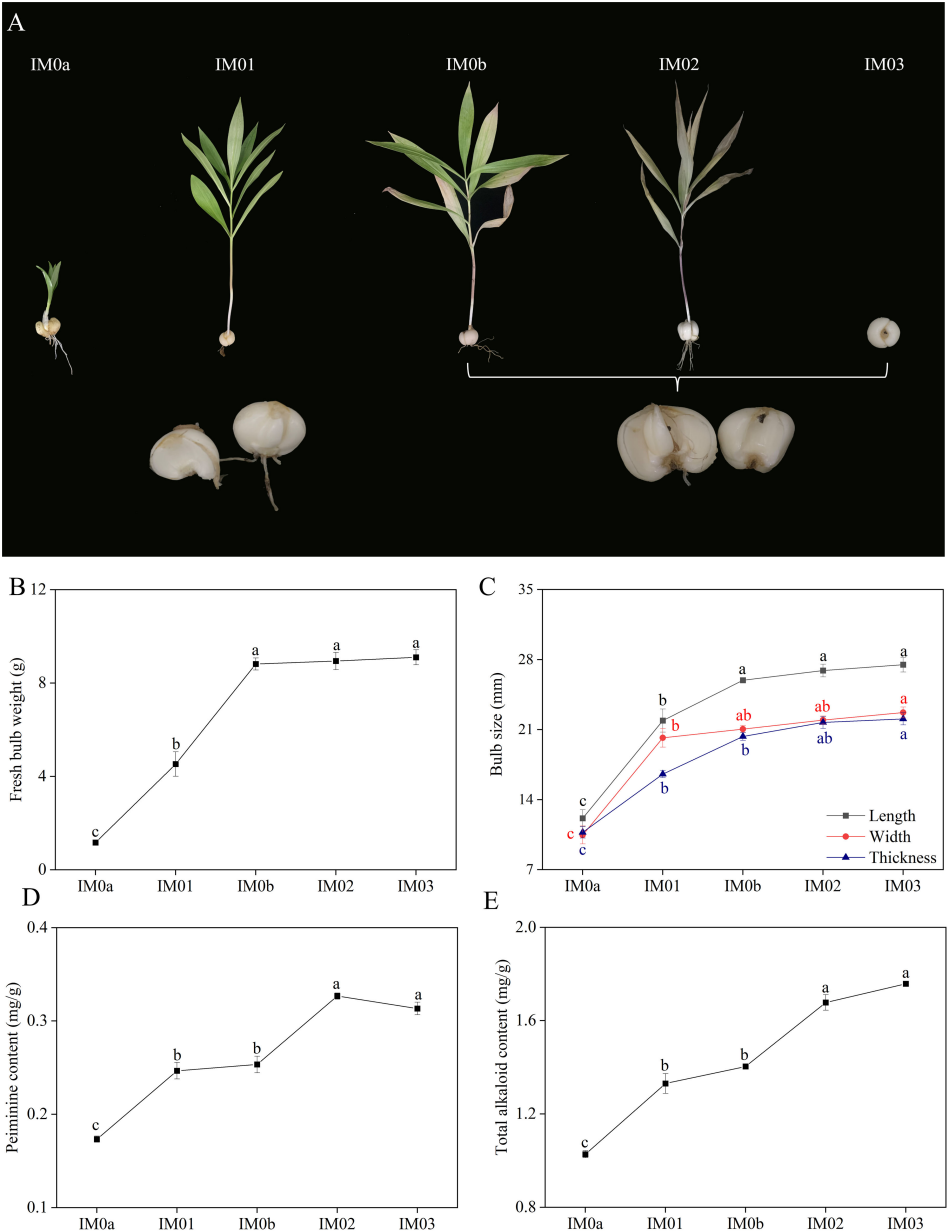
Dynamic phenotypic and biochemical variations in regenerated bulb development of *F. hupehensis*. **(A)**. Phenotypic changes in (*F*) *hupehensis* at five developmental stages. **(B)**. Bulb size (length, width, and thickness) changes. **(C)**. Details of changes in fresh bulb weight. **(D)**. Details of changes in peiminine content. **(E)**. Details of changes in total alkaloid content. All data in the Figure are represented as mean ± standard error. Different letters in each system represent significant differences at *p<*0.05.

Phenotypic and biochemical changes, including fresh bulb weight, bulb size, peiminine content, and total alkaloid content, were investigated in the regenerated bulbs at five different stages (IM0a, IM01, IM0b, IM02, and IM03) (**Figure 2A**). The fresh bulb weight of *F. hupehensis* sharply increased from IM01a to IM01b, but slightly increased from IM0b to IM03, reaching 9.10 g at IM03 (**Figure 2B**). The length, width, and thickness of bulbs enormously increased from IM0a (12.13 mm, 10.47 mm, and 10.72 mm, respectively) to IM01 (21.91 mm, 20.18 mm, and 16.55 mm, respectively), and then gradually increased from IM01 to IM03, reaching their maximum at IM03 (27.49 mm, 22.71 mm, and 22.08 mm, respectively) (**Figure 2C**). The peiminine and total alkaloid contents significantly increased from IM0a (0.17 mg/g and 1.03 mg/g, respectively) to IM01 (0.25 mg/g and 1.33 mg/g, respectively) (*p*<0.05), reaching their maximum at IM03 (0.33 mg/g) and IM02 (1.76 mg/g), respectively. Moreover, their contents were not significantly different between IM01 and IM0b, or between IM02 and IM03 (**Figures 2D, E**). The results showed that the size and weight of fresh bulbs increased significantly from IM0a to IM0b, whereas the alkaloids increased significantly from IM0a to IM01 and IM0b to IM02, respectively.

### 3.2 Changes in alkaloid metabolites with regenerated bulb development of *F. hupehensis*

**Figure 3:**
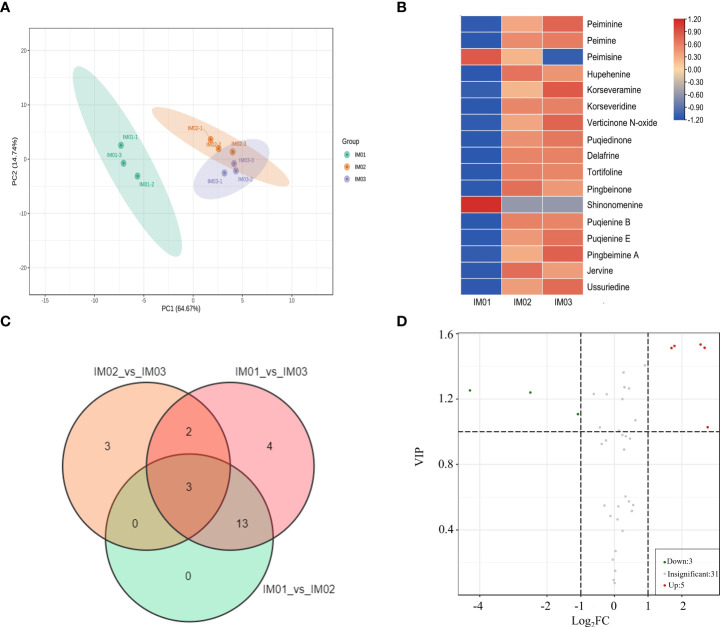
Metabolite analysis in regenerated bulbs of *F. hupehensis*. **(A)**. PCA of metabolomic data. **(B)**. Heatmap of DEAs. **(C)**. Venn diagram of DEAs between the three compared groups: “IM01_vs_IM02”, “IM01_vs_IM03”, and “IM02_vs_IM03”. **(D)**. Volcano plots of DEAs in the comparison group of “IM02_vs_IM03”.

To investigate the alkaloid metabolites in regenerated bulb development of *F. hupehensis*, we performed targeted metabolomic identification using UPLC-MS at three stages (IM01, IM02, and IM03). Pearson’s correlation analysis confirmed the authenticity of the metabolomic data (**Figure S1**). PCA revealed that the samples within the IM01 stage group clustered together, whereas the IM02 and IM03 stage samples failed to separate (**Figure 3A**). The alkaloid metabolites are listed in **Table S2**, and steroidal alkaloids were the most common DEAs in the regenerated bulb development of *F. hupehensis* (**Table S3**). In total, 17 significantly differentially expressed steroidal alkaloids were identified (**Figure 3B**). Interestingly, compared with IM01, the levels of peiminine, peimine, hupehenine, korseveramine, korseveridine, hericenone N-oxide, puqiedinone, delafrine, tortifoline, pingbeinone, puqienine B, puqienine E, pingbeimine A, jervine, and ussuriedine significantly increased in IM02 and IM03, whereas peimisine and shinonomenine decreased. The upregulated differentially expressed steroidal alkaloids accounted for 88.24%, indicating that steroidal alkaloids accumulated during the development of regenerated bulbs. Moreover, the DEAs in the “IM01_vs_IM02”, “IM01_vs_IM03”, and “IM02_vs_IM03” groups were analyzed, and there were 16, 22, and 8 DEAs in each of these groups, respectively (**Figure 3C**). Three alkaloid metabolites (4-hydroxymandelonitrile, p-coumaroylputrescine, and N-feruloylputrescine) were common among these groups (**Tables S4–S6**). Compared to IM02, the levels of five DEAs (indole, N-benzylformamide, 4-hydroxymandelonitrile, 3-indolepropionic acid, and 1-methoxy-indole-3-acetamide) were upregulated in IM03, whereas those of three DEAs (p-coumaroylputrescine, N-feruloylputrescine, and peimisine) were downregulated in IM03 (**Figure 3D**; **Table S6**). Twenty percent of alkaloids, of which only one was a steroidal alkaloid (peimisine), were altered significantly in IM03 compared to IM02, suggesting that the accumulation of steroidal alkaloids occurred before IM02.

### 3.3 Changes in gene expression with regenerated bulb development of *F. hupehensis*

RNA-Seq analysis was performed on the IM01, IM02, and IM03 samples, and the results are listed in **Table S7**. In general, 473.71, 473.22, and 476.88 million raw reads with 433.04, 445.39, and 458.73 million clean reads were obtained from IM01, IM02, and IM03 groups, respectively. The average GC content of IM01, IM02, and IM03 was 51.30%, 50.33%, and 50.69%, respectively. Moreover, the Q20 values were 97.18%, 97.22%, and 97.22%, and the Q30 values were 92.33%, 92.35%, and 92.94% for the IM01, IM02, and IM03 groups, respectively, indicating good quality transcriptome sequencing. A total of 103,545 transcripts and 90,032 unigenes were generated (**Table S8**) with mean lengths of 839 and 928 bp, respectively. The N50 and N90 values of these transcripts were 1336 and 331 bp, respectively, whereas the unigene N50 and N90 values were 1359 and 390 bp, respectively. Thus, the quality of transcriptome assembly was satisfactory.

**Figure 4:**
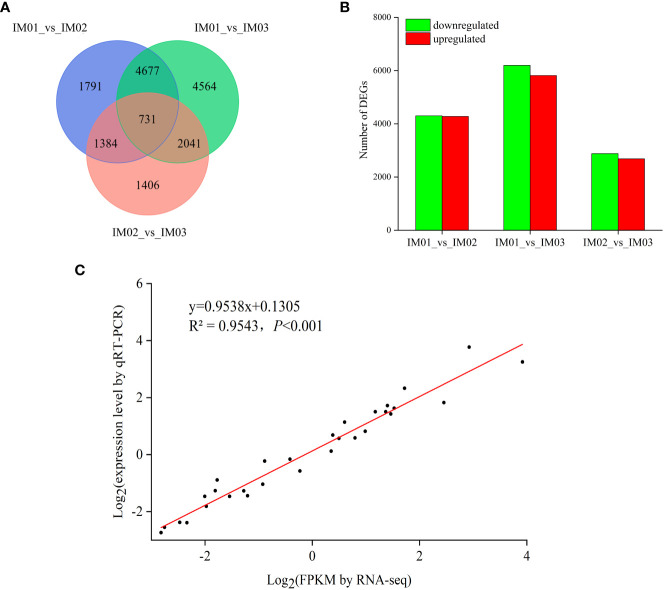
DEGs in regenerated bulbs of *F. hupehensis*. **(A)**. Venn diagram of DEGs for different comparisons: “IM01_vs_IM02”, “IM01_vs_IM03”, and “IM02_vs_IM03”. **(B)**. The number of up- and downregulated genes for different comparisons. **(C)**. Linear regression of RNA-Seq and qRT-PCR data that are expressed as a log_2_ fold change.

The differential expression of unigenes was analyzed during regenerated bulb development in *F. hupehensis*. Comparing “IM01_vs_IM02”, “IM01_vs_IM03”, and “IM02_vs_IM03”, 8,583 (4,279 upregulated and 4,304 downregulated), 12,013 (5,817 upregulated and 6,196 downregulated), and 5,562 (2,684 upregulated and 2,878 downregulated) significant DEGs were identified, respectively (**Figures 4A, B**). The number of upregulated and downregulated DEGs for “IM01_vs_IM03” was the largest, followed by “IM01_vs_IM02”. Notably, the number of up- and downregulated DEGs for “IM02_vs_IM03” was the lowest compared with the other groups. The results showed that gene expression levels at these three stages in the regenerated bulb development of *F. hupehensis* were significantly different.

To validate the RNA-Seq results, DEGs were randomly selected for qRT-PCR analysis (**Table S9**). As expected, the expression levels of these genes determined using qRT-PCR were consistent with the RNA-Seq results (**Table S10**). Pearson’s correlation analysis confirmed the reliability of RNA-Seq data (**Figure S2**). Moreover, the correlation coefficient (R^2^= 0.9543, *p*<0.001) validated that the RNA-Seq data were duplicable and reliable (**Figure 4C**; **Table S11**).

Based on KEGG enrichment analysis, the top 20 enriched pathways are shown in the bubble diagram (**Figure S3**). Comparing “IM01_vs_IM02”, the DEGs were mainly enriched in “metabolic pathways”, “biosynthesis of secondary metabolites”, “protein processing in endoplasmic reticulum”, “endocytosis”, and “fatty acid biosynthesis”. For “IM01_vs_IM03”, “metabolic pathways” and “protein processing in the endoplasmic reticulum” were the two main enriched pathways. In the case of “IM02_vs_IM03”, the DEGs were mainly enriched in “protein processing in endoplasmic reticulum”. Notably, the biosynthesis of secondary metabolites was significantly enriched for “IM01_vs_IM02” (*p*<0.05), indicating that the accumulation of secondary metabolites mainly occurred before IM02.

### 3.4 Differentially expressed genes in the steroidal alkaloid biosynthesis pathway

**Figure 5:**
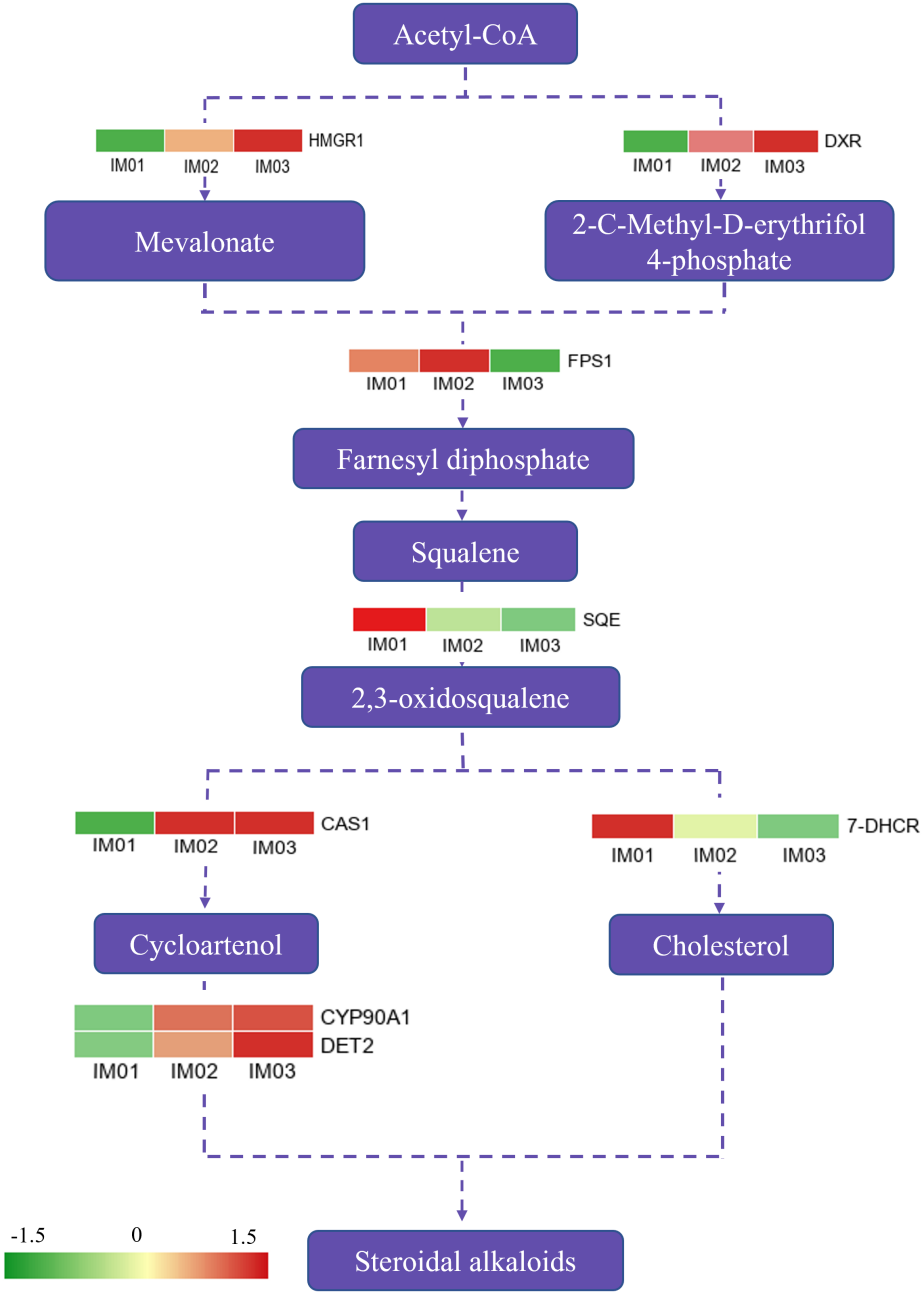
DEGs in proposed steroidal alkaloid pathway.

A possible steroidal alkaloid biosynthesis pathway diagram was constructed according to previous reports (Kumar et al., 2021; Sharma et al., 2021), and DEGs in this pathway were identified (**Figure 5**; **Table S12**). In total, eight DEGs were mapped to this pathway. Compared to IM01, five genes (*HMGR1*, *DXR*, *CAS1*, *CYP 90A1*, and *DET2*) were significantly upregulated in IM02 and IM03, whereas the *SQE* and *7-DHCR* were downregulated. *FPS1* was expressed at a higher level in IM02, whereas it was expressed at a lower level in IM03 than in IM01. Notably, compared with IM01, six out of eight genes were expressed at higher levels in IM02 and IM03, similar to the changes in the majority of differential steroidal alkaloids.

### 3.5 Co-expression network analysis of genes associated with steroidal alkaloid biosynthesis

**Figure 6:**
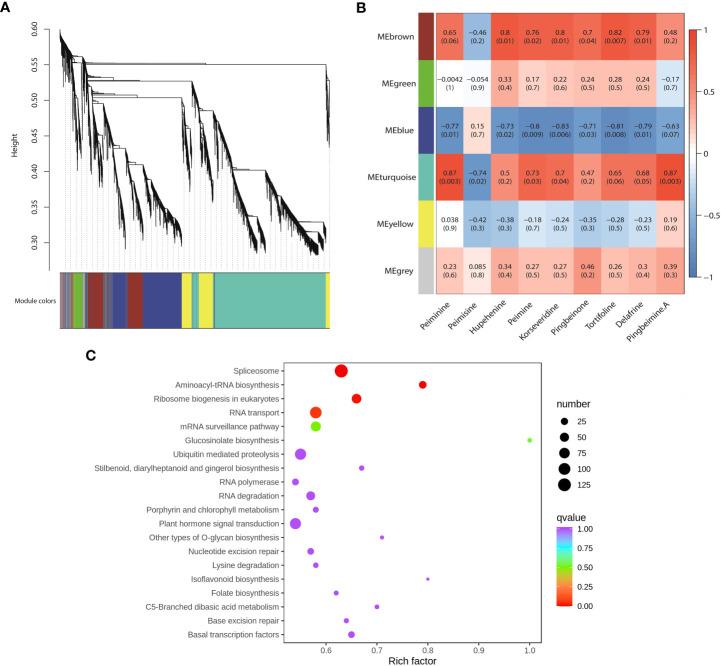
WGCNA of DEGs related to steroidal alkaloids. **(A)**. Cluster dendrogram. **(B)**. Correlations between modules and steroidal alkaloids. The number represents the correlation coefficient and *p* value. Blue indicates low correlation, and red indicates high correlation. **(C)**. KEGG analysis of 4,268 genes in the MEturquoise module.

To uncover the gene regulatory network of steroidal alkaloid biosynthesis in *F. hupehensis*, WGCNA was conducted using 8,985 genes with FPKM>5. In total, six distinct gene expression modules, labeled with different colors, were identified (**Figure 6A**). Furthermore, the correlation coefficients between the modules and nine steroidal alkaloids (peiminine, peimisine, hupehenine, peimine, pingbeimine A, korseveridine, pingbeinone, tortifoline, and delafrine) were calculated, and the heatmaps are shown in **Figure 6B**. Among the six modules, 1,238 genes in the MEbrown module were significantly positively correlated with hupehenine (r=0.80, *p*<0.05), peimine (r=0.76, *p*<0.05), korseveridine (r=0.80, *p*<0.05), pingbeinone (r=0.70, *p*<0.05), tortifoline (r=0.70, *p*<0.01), and delafrine (r=0.80, *p*<0.05). However, 2,256 genes in the MEblue module were significantly negatively correlated with peiminine (r=-0.77, *p*<0.05), hupehenine (r=-0.73, *p*<0.05), peimisine (r=-0.80, *p*<0.01), korseveridine (r=-0.83, *p*<0.01), pingbeimine A (r=-0.71, *p*<0.05), tortifoline (r=-0.81, *p*<0.01), and delafrine (r=-0.63, *p*<0.05). Interestingly, the MEturquoise module with the highest number of genes (4,268) was significantly positively correlated with peiminine (r=0.87, *p*<0.01), peimine (r=0.73, *p*<0.05), korseveridine (r=0.70, *p*<0.05), and pingbeimine A (r=0.87, *p*<0.01), and negatively correlated with peimisine (r=-0.74, *p*<0.05). According to the top 20 KEGG pathways, DEGs were associated with the biosynthesis of steroidal alkaloids in the MEturquoise module (**Figure 6C**). Moreover, the “RNA transporter”, “C5 branched dibasic acid metabolism”, and “basal transcription factors” were involved, indicating that DEGs enriched in these three pathways may regulated the biosynthesis of peiminine, peimine, korseveridine and pingbeimine A.

**Figure 7:**
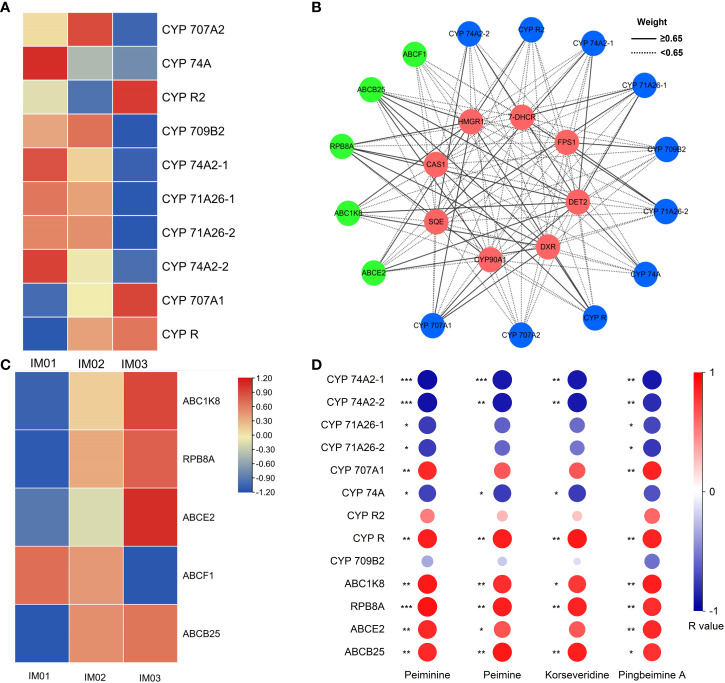
CYP, and ABC gene analysis. **(A)**. Heatmap of CYP genes in the MEturquoise module. **(B)**. Co-expression network analysis of eight candidate genes, CYP, and ABC transporter genes in the MEturquoise module. The lines represent the weighted Pearson’s correlation coefficients. Green, blue, and red denote ABC transporters, CYPs, and candidate genes, respectively. **(C)**. Heatmap of genes encoding ABC transporters in the MEturquoise module. **(D)**. Heatmap of CYP and ABC transporter genes with weighted Pearson’s correlation coefficient values ≥6.5 and steroidal alkaloids related to the MEturquoise module, r’s value were calculated (*p<0.05; **p<0.01; ***p<0.001).

In the MEturquoise module, ten CYP and five ABC transporter genes expressed at high levels (FPKM≥10) were identified. Five (50.0%), two (20.0%), and three (30.0%) genes encoding CYPs were maximally expressed in IM01, IM02, and IM03, respectively (**Figure 7A**; **Table S13**). One (20.0%) and four (80.0%) genes encoding ABC transporters were maximally expressed in IM01 and IM03, respectively (**Figure 7C**; **Table S13**). The potential interaction network diagram of the DET2, CYP, and ABC transporter genes in the MEturquoise module is plotted and visualized in **Figure 6B**. In this network diagram, nine CYP genes (*CYP 74A2-1*, *CYP 74A2-2*, *CYP 71A26-1*, *CYP 71A26-2*, *CYP 707A1*, *CYP 74A, CYP R2*, *CYP R*, and *CYP 709B2*) and four ABC transporter genes (*RPB8A*, *ABCB25*, *ABCE2*, and *ABC1K8*) were highly correlated with *CAS1*, *7-DHCR*, *HMGR1*, *DXR*, *FPS1*, *SQE*, *CYP90A1*, and *DET2* identified in steroidal alkaloid biosynthesis (**Figure 7B**; **Table S14**). Subsequently, Pearson’s r values between these highly expressed genes and the four steroidal alkaloids related to the MEturquoise module (peiminine, peimine, korseveridine, and pingbeimine A) were calculated (**Figure 7D**). *CYP 74A2-1* and *CYP 74A2-2* were significantly negatively correlated with peiminine, peimine, korseveridine, and pingbeimine A (|R|>0.8, *p*<0.01), whereas *CYP R* was positively correlated (R>0.85, *p*<0.01). *CYP 71A26-1*, *CYP 71A26-2*, and *CYP74A* were negatively correlated with peiminine and pingbeimine A (|R|>0.7, *p*<0.05), whereas *CYP707A1* was positively correlated (R>0.8, *p*<0.01). *ABC1K8, RPB8A*, and *ABCB25* were strongly and positively correlated with peiminine, peimine, korseveridine, and pingbeimine A (R>0.8, *p*<0.05). *ABCE2* strongly and positively correlated with peiminine, peimine, and pingbeimine A (R>0.8, *p*<0.05). The results showed that the expression of *CYP 74A2-1*, *CYP 74A2-2*, *CYP R*, *CYP 71A26-1*, *CYP 71A26-2*, *CYP74A*, and *CYP707A1* genes may play a complex role in the accumulation of steroidal alkaloids. *ABC1K8, RPB8A*, and *ABCB25* may positively regulate steroidal alkaloid biosynthesis.

### 3.6 Transcription factors involved in steroidal alkaloid biosynthesis

**Figure 8:**
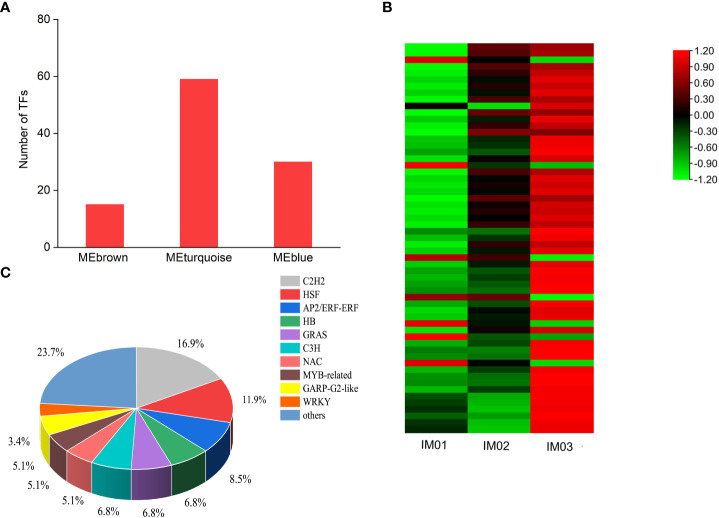
TFs analysis based on WGCNA. **(A)**. TFs in the MEbrown, MEturquoise, and MEblue modules. **(B)**. Heatmap of TFs in the MEturquoise module. **(C)**. Classifications and proportions of TFs in the MEturquoise module.

Based on the WGCNA, 15, 59, and 30 transcription factors (TFs) were identified in the MEbrown, MEturquoise, and MEblue modules, respectively (**Figure 8A**). Furthermore, a heatmap of TFs in the MEturquoise module was constructed (**Figure 8B**). In the MEturquoise module, 7 (11.86%) and 52 (88.14%) TF genes were maximally expressed in IM01 and IM03, respectively, suggesting that the bulk of highly expressed TFs may positively regulate the biosynthesis of steroidal alkaloids in the ripening stage (**Figure 7B**; **Table S15**). A total of 59 TF genes were classified into 22 families (**Table S16**), including 10 (16.90%) C2H2s, 7 (11.90%) HSFs, 5 (8.50%) AP2/ERFs, 4 (6.80%) HBs, 4 (6.80%) GRASs, 4 (6.80%) C3Hs, 3 (5.10%) NACs, 3 (5.10%) MYB-related TFs, 3 (5.10%) GARP-G2-like TFs, 2 (3.40%) WRKYs, and 14 (23.7%) other TFs.

## 4 Discussion

The phenotype and predominant biochemical ingredients in *F. hupehensis* usually change during regenerated-bulb development. Investigating changes in phenotype and predominant biochemical ingredients is beneficial for determining the appropriate harvest period for regenerated bulbs. In this study, fresh bulb weight and size were measured during regenerated bulb development of *F. hupehensis*. The regenerated bulbs enlarged sharply from IM0a to IM0b, reaching a maximum at IM03 (**Figures 2B, C**). Peiminine is the main biochemical component determining the quality of *F. hupehensis*. In this study, peiminine and total alkaloid contents were measured during the development of regenerated bulbs. The results showed that the peiminine and total alkaloid contents accumulated rapidly from IM0a to IM01 and from IM0b to IM02, reaching a maximum at IM02 and IM03, respectively (**Figures 2D, E**). In particular, the phenotype and predominant biochemical ingredients were not significantly different between IM02 and IM03, indicating that regenerated bulbs of *F. hupehensis* could be harvested in early June or July.

Previous studies on the detection of alkaloids in *F. hupehensis* have been limited to a few alkaloids (Zhang et al., 2007; Zhang et al., 2008; Che et al., 2020). Widely targeted metabolomics has been effectively applied to metabolite identification (Wang, H. et al., 2021; Hu et al., 2022a). In the present study, a targeted alkaloid metabolomics approach was used to provide more comprehensive insight into alkaloid variations during regenerated bulb development. Similar to our previous report (Duan et al., 2022), 40 alkaloids were identified at different stages in the present study, with steroidal alkaloids being the largest alkaloid class. Previous studies have shown that steroidal alkaloids are the most effective chemical substances in *F. hupehensis* (Liu et al., 2021). In this study, the steroidal alkaloids were the most common DEAs in the regenerated bulb development of *F. hupehensis*. The majority of differentially expressed steroidal alkaloids were upregulated in IM02 and IM03, indicating that steroidal alkaloids were accumulated during the development of regenerated bulbs. However, only one steroidal alkaloid (peimisine) was significantly altered in IM03 compared with IM02, whereas other steroidal alkaloid metabolites did not differ significantly, suggesting that the accumulation of steroidal alkaloids occurred before IM02. The steroidal alkaloids identified during the ripening stages (IM02 and IM03) reflect the chemical basis of *F. hupehensis* medicinal composition to a certain degree. In addition, indole, N-benzylformamide, 4-hydroxymandelonitrile, 3-indolepropionic acid, and 1-methoxy-indole-3-acetamide were upregulated in IM03 compared with IM02, whereas p-coumaroylputrescine, and N-feruloylputrescine were downregulated, and these significantly altered alkaloid metabolites with significantly altered levels in IM03 need to be further explored.

The regenerated bulb development process involves a series of gene expressions and biological and metabolic pathways. Transcriptome sequencing has been widely used to identify the key genes regulating the accumulation of steroidal alkaloids in *Fritillaria* and to explore the molecular mechanisms of fruit ripening in plants (Onik et al., 2018; Wang, H. et al., 2021; Duan et al., 2022). To dissect the developmental mechanism of regenerated bulbs, transcriptomic analysis of regenerated bulbs of *F. hupehensis* from three stages (IM01, IM02, and IM03) was conducted. KEGG enrichment analysis indicated that the accumulation of secondary metabolites mainly occurred prior to IM02. It is widely known that alkaloids are important secondary metabolites (Kaur and Arora, 2015). The KEGG analysis results were consistent with those of the metabolome analysis, further validating that the accumulation of steroidal alkaloids occurred before IM02. However, the steroidal alkaloid pathway and the functional characterization of its regulatory genes remain limited (Zhao et al., 2018). As described in our previous study (Duan et al., 2022), the 2-C-methyl-d-erythritol 4-phosphate (MEP) and Mevalonate (MVA) pathways could be the main pathways for the accumulation of isopentenyl diphosphate (IPP) to biosynthesize steroidal alkaloids (**Figure 5**). HMG-CoA reductase (HMGR) and 1-deoxy-D-xylulose-5-phosphate reductoisomerase (DXR) have been reported to be the key enzyme MVA and MEP pathways, respectively (Valitova et al., 2016; Zolfaghari et al., 2020). In this study, one *HMGR1* and one *DXR* gene were identified in the process of steroidal backbone biosynthesis (**Figure 5**), and they were upregulated in IM02 and IM03. Farnesyl diphosphate synthase (FPS) supplies sesquiterpene precursors for farnesylation and geranylgeranylation of proteins in isoprenoid biosynthesis (Szkopińska and Płochocka, 2005). In the current study, one *FPS1* gene was expressed maximally in IM02 compared with IM01 and IM03. Squalene epoxidase (SQE) is an essential enzyme to convert squalene to 2,3-oxidosqualene (Kumar et al., 2021), and 2, 3-oxidosqualene is cyclized by cycloartenol synthase (CAS) to produce cycloartenol, which serves as an important precursor of steroidal alkaloids (Wang et al., 2019; Lu et al., 2022). Subsequently, steroidal alkaloids are produced by modification reactions (Duan et al., 2022). In the present study, one *CAS1* gene, one *CYP 90 A1* gene, and one *DET* gene were expressed at higher levels in IM02 and IM03 than in IM01, which might lead to the production of peiminine, peimine, hupehenine, korseveramine, korseveridine, hericenones N-oxide, puqiedinone, delafrine, tortifoline, pingbeinone, puqienine B, puqienine E, pingbeimine A, jervine, and ussuriedine in the bulb development of *F. hupehensis*. In addition, 7-dehydrocholesterol reductase (7-DHCR) is a key enzyme that catalyzes the production of another cholesterol precursor (Wang et al., 2021b). In our study, one *SQE* gene and one *7-DHCR* gene were downregulated in IM02 and IM03 compared with IM01. Thus, we hypothesized that the downregulation of *FPS1*, *SQE* and *17-DHCR* may play an essential role in peimisine biosynthesis in *F. hupehensis*. Remarkably, five out of eight genes were highly expressed in IM02 and IM03, which was largely consistent with the changes in the majority of differential steroidal alkaloids.

WGCNA is an effective tool for identifying key genes involved in a particular process and exploring metabolic mechanisms (Xu et al., 2020; Wu et al., 2022). It has been proved meaningfully in petal band-specific coloration mechanism (Hao et al., 2020), sugar and hormone accumulation mechanisms under low-temperature-stress (Wu et al., 2022), and flavonoid and amino acid synthesis mechanisms (Hu et al., 2022a; Hu et al., 2022b). In the present study, WGCNA was performed to elucidate steroidal alkaloid biosynthesis in *F. hupehensis*. Finally, three modules were identified to be significantly correlated with steroidal alkaloids, and the MEturquoise module was significantly positively correlated with peiminine, peimine, korseveridine, and pingbeimine A, but negatively correlated with peimisine (**Figure 6B**). Moreover, the DEGs in the MEturquoise module were enriched in “RNA transporter”, “C5 branched dibasic acid metabolism”, and “basal transcription factors”, indicating that DEGs enriched in these three pathways may regulate the biosynthesis of peiminine, peimine, korseveridine, and pingbeimine A (**Figure 6C**).

As the largest monooxygenases in plants, CYPs play an essential role in steroidal alkaloid biosynthesis (Sun et al., 2011; Sharma et al., 2021). Kumar et al. (2021) reported that ABC transporters participate in steroidal sipeimine biosynthesis. In the present study, ten genes encoding CYPs and five genes encoding ABC transporters expressed at high levels (FPKM≥10) were identified in the MEturquoise module. Furthermore, nine CYP genes (*CYP 74A2-1, CYP 74A2-2, CYP 71A26-1, CYP 71A26-2, CYP 707A1, CYP 74A, CYP R2, CYP R, and CYP 709B2*) and four ABC transporter genes (*RPB8A*, *ABCB25*, *ABCE2*, and *ABC1K8*) were highly correlated with *CAS1*, *7-DHCR*, *HMGR1*, *DXR*, *FPS1*, *SQE*, *CYP90A1*, and *DET2* genes identified in steroidal alkaloid biosynthesis, indicating that these genes may play an essential role in steroidal alkaloid biosynthesis in *F. hupehensis*. Moreover, the significant correlation between the expression of these nine CYP and four ABC transporter genes and the accumulation of peiminine, peimine, korseveridine, and pingbeimine A further validated their important role in the synthesis of peiminine, peimine, korseveridine, and pingbeimine A. Notably, *CYP 74A2-1* and *CYP 74A2-2* may play a negative role in the biosynthesis of peiminine, peimine, korseveridine, and pingbeimine A, whereas *CYP R* plays a positive role. *CYP 71A26-1*, *CYP 71A26-2*, and *CYP74A* were negatively correlated with peiminine and pingbeimine A levels, whereas *CYP707A1* levels were positively correlated. *ABC1K8, RPB8A*, and *ABCB25* were strongly positively correlated with peiminine, peimine, korseveridine and pingbeimine A levels, whereas *ABCE2* was strongly and positively correlated with peiminine, peimine, and pingbeimine A levels. These results show that CYPs perform a distinct and complicated function, and ABC transporters play a positive role in the steroidal alkaloid accumulation. Further verification is required to determine their functions in the biosynthesis of peiminine, peimine, korseveridine, and pingbeimine A.

Prominent regulators of TFs play crucial roles in the transcriptional regulation of secondary metabolite biosynthesis (Meraj et al., 2020; Prakash et al., 2023). Increasing evidence has shown that TFs, including AP2/ERF, bHLH, WRKY, and MYB families, are involved in the biosynthesis of alkaloids (Chen et al., 2019; Godbole et al., 2022). Recently, Lu et al. (2022) identified several AP2/ERF, bHLH, MYB, C2H2, and bHLH TFs that directly or indirectly regulate steroidal alkaloids in *Fritillariae cirrhosae*. In this study, TFs identified in the MEbrown, MEturquoise, and MEblue modules were counted and TFs in the MEturquoise module were analyzed. The results showed that the bulk of highly expressed C2H2, HSF, AP2/ERF, HB, GRAS, C3H, NAC, MYB-related TFs, GARP-G2-like TFs, and WRKY may positively regulate steroidal alkaloid biosynthesis during bulb development in *F. hupehensis*. However, TFs are comprehensive, complex, and multiplex, and their functional characterizations should be identified using yeast one-hybrid, dual-luciferase, and biochemical assays in future studies.

## 5 Conclusion

This study found that regenerated bulbs of *F. hupehensis* could be harvested in early June or July. Metabolomic analysis showed that steroidal alkaloids accumulated before IM02 (early June). Simultaneously, KEGG enrichment analysis further validated the biosynthesis of secondary metabolites (steroidal alkaloids) that occurred before early June. Eight genes that were differentially expressed in the steroidal alkaloid biosynthesis pathway were identified. WGCNA identified four ABC transporter genes (*RPB8A*, *ABCB25*, *ABCE2*, and *ABC1K8*) that might play a positive role in the accumulation of peiminine, peimine, korseveridine, and pingbeimine A and nine CYP genes (*CYP 74A2-1*, *CYP 74A2-2*, *CYP 71A26-1*, *CYP 71A26-2*, *CYP 707A1*, *CYP 74A, CYP R2*, *CYP R*, and *CYP 709B2*). Moreover, high expression of C2H2, HSF, AP2/ERF, HB, GRAS, C3H, NAC, MYB-related TFs, GARP-G2-like TFs, and WRKY may play a positive role in the accumulation of peiminine, peimine, korseveridine, and pingbeimine A. This study may be beneficial for unraveling steroidal alkaloid biosynthesis mechanisms in the regenerated bulb development of *F. hupehensis*. These findings provide a theoretical foundation and technical support for the sustainable cultivation and clinical application of *F. hupehensis*.

## Data availability statement

The original contributions presented in the study are publicly available. This data can be found here: https://ngdc.cncb.ac.cn/gsa/browse/CRA009125.

## Author contributions

JG framed the experimental design and approved the final version of the manuscript. YD performed the experiments, analyzed transcriptomic and metabolomic data, and wrote the manuscript. JY analyzed metabolomic and transcriptomic data and revised the manuscript. JW, FW, and KZ helped collect the experimental samples and perform the experiments. XG and SM participated in plot selection and guided the implementation of the field trials. TT collected the experimental samples and revised the manuscript. All authors contributed to the article and approved the submitted version.
