# Supplementary material for: Transcriptomic and metabolomic analyses provide new insights into the appropriate harvest period in regenerated bulbs of Fritillaria hupehensis
Source: Front Plant Sci. 2023 Feb 15;14:1132936. doi: 10.3389/fpls.2023.1132936 (PMC9975545; doi:10.3389/fpls.2023.1132936)
Supplement: Supplementary file 1 [file DataSheet_1.docx]

Supplementary Material

# Supplementary Figures


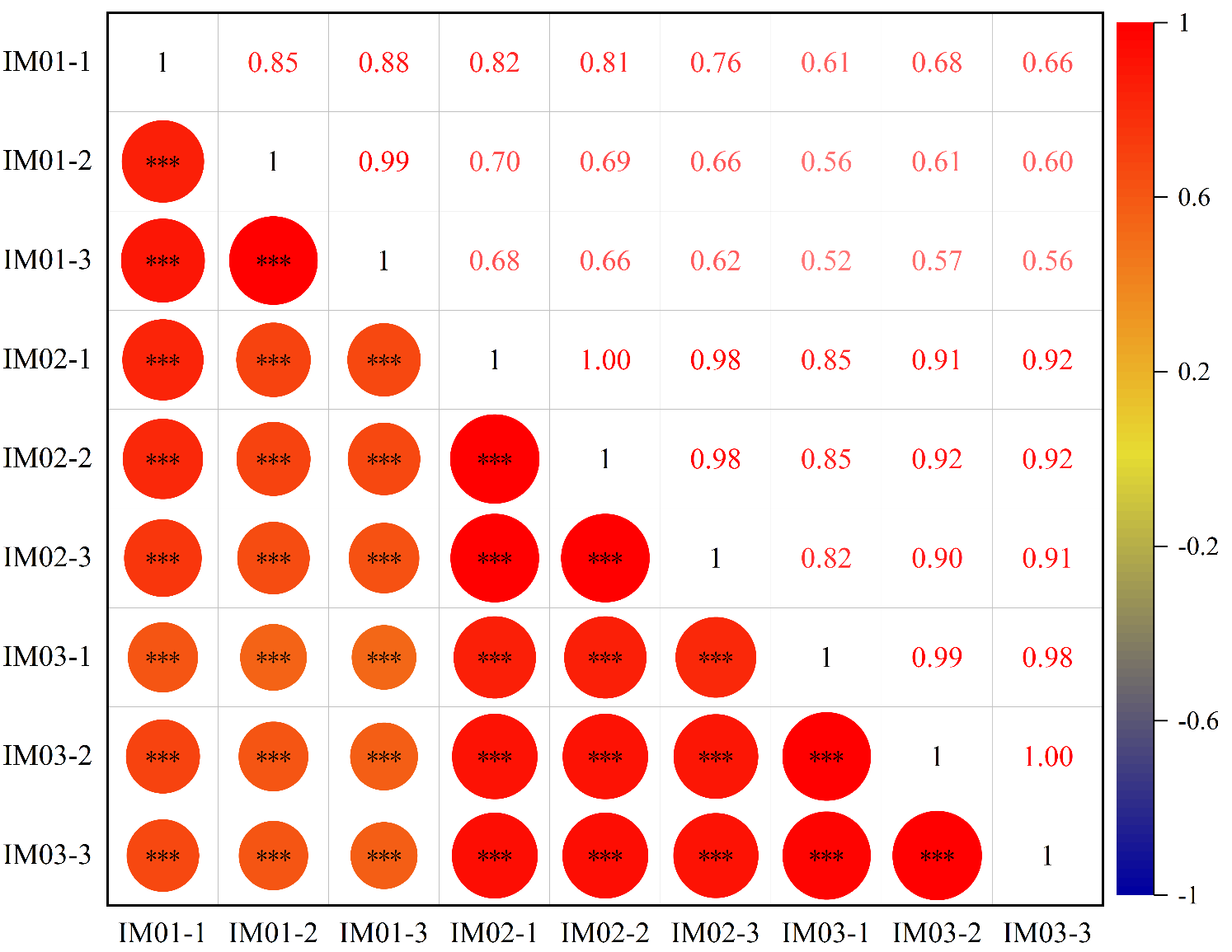


**Supplementary Figure 1** Pearson’s correlation analysis of metabolomic data.


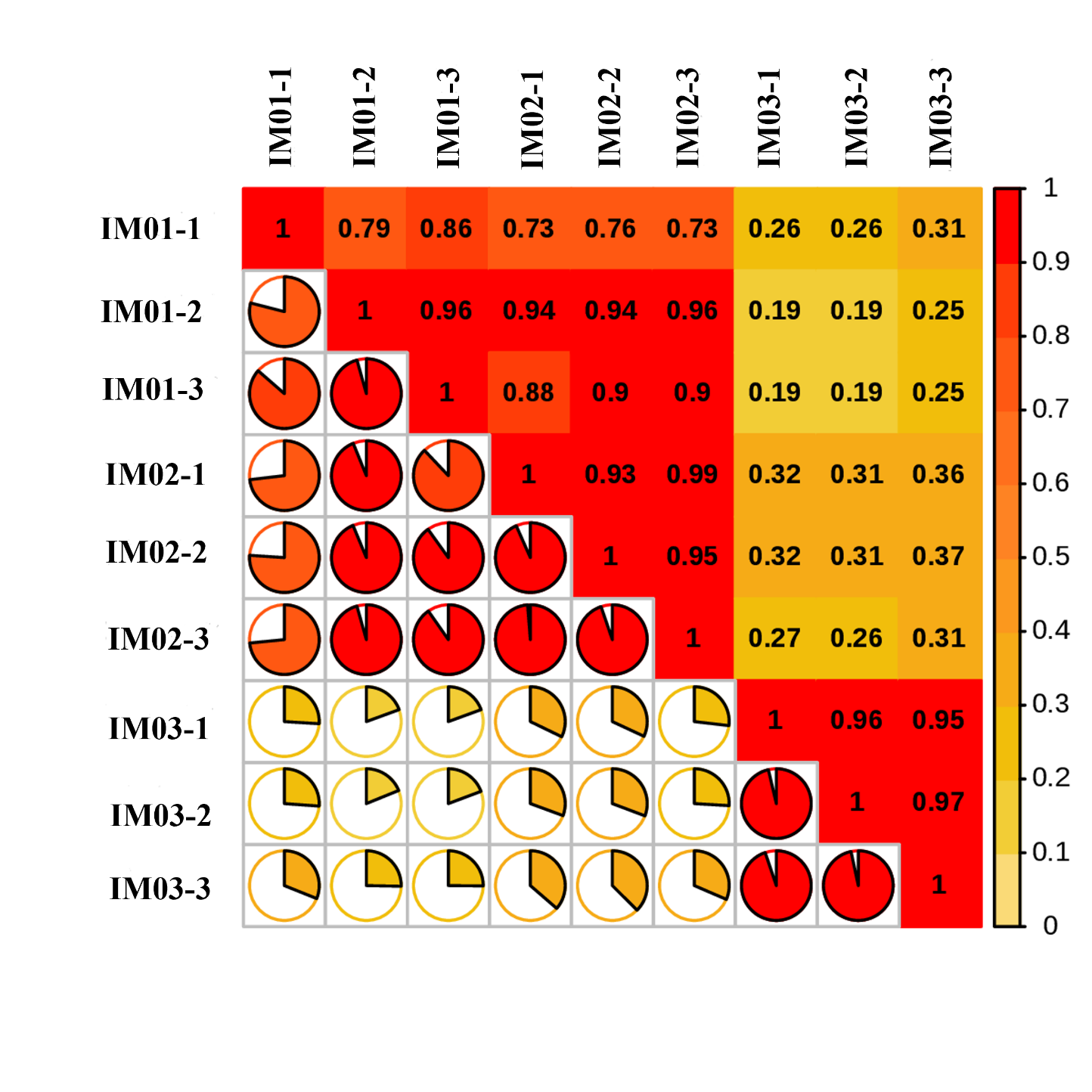


Supplementary Figure 2. Pearson’s correlation analysis of RNA-Seq data


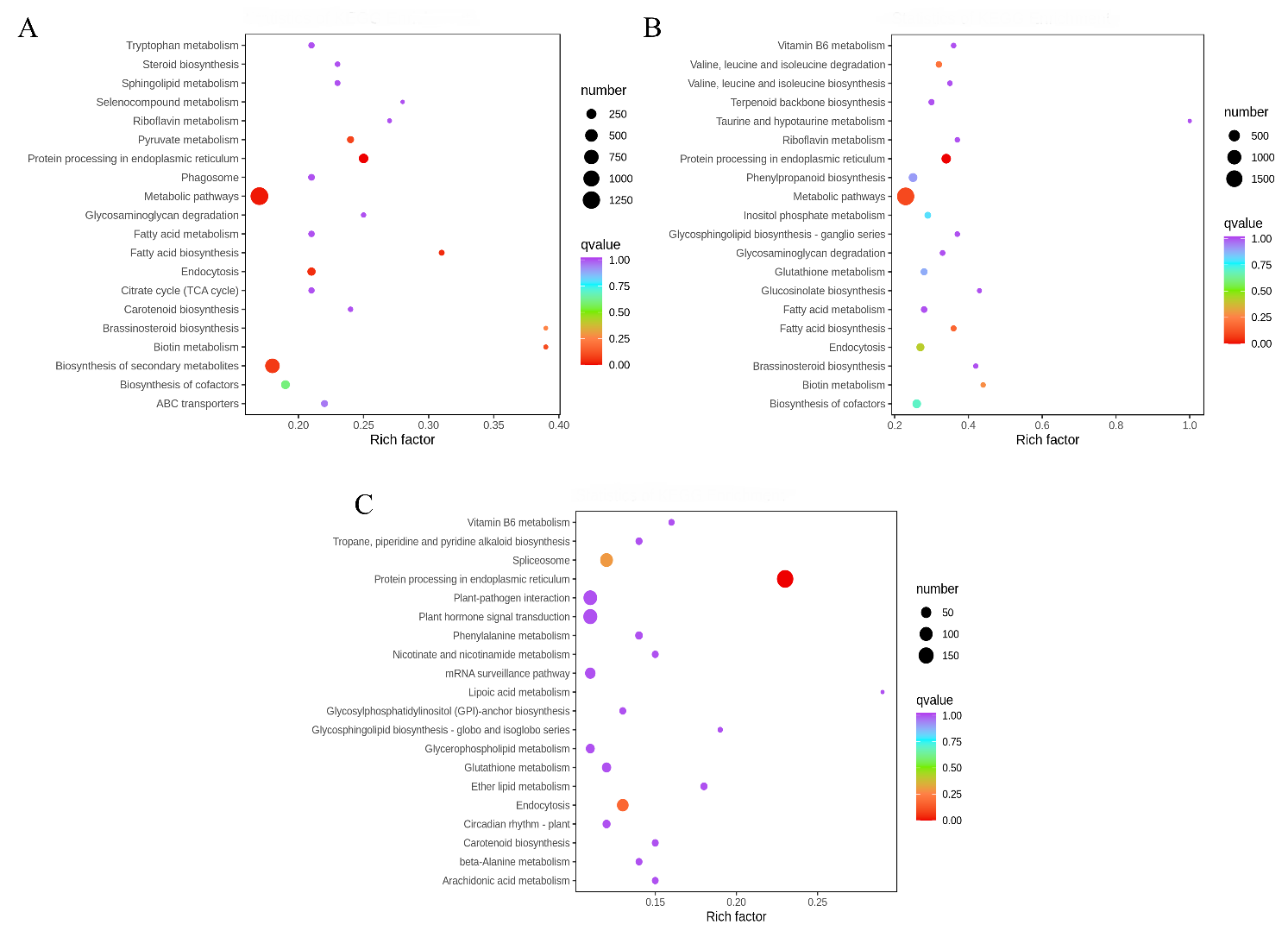


**Supplementary Figure 3** KEGG enrichment analysis of three groups. (A) “IM01_vs_IM02”. (B)IM01_vs_IM03”. (C) “IM02_vs_IM03”.
